# Soluble CD40L activates soluble and cell-surface integrin αvβ3, α5β1, and α4β1 by binding to the allosteric ligand-binding site (site 2)

**DOI:** 10.1016/j.jbc.2021.100399

**Published:** 2021-02-09

**Authors:** Yoko K. Takada, Michiko Shimoda, Emanual Maverakis, Brunie H. Felding, R. Holland Cheng, Yoshikazu Takada

**Affiliations:** 1 Department of Dermatology, UC Davis School of Medicine, Sacramento, California, USA; 2 Department of Biochemistry and Molecular Medicine, UC Davis School of Medicine, Sacramento, California, USA; 3 The Scripps Research Institute, Department of Molecular and Experimental Medicine, La Jolla, California, USA; 4 Department of Molecular and Cellular Biology, UC Davis, Davis, California, USA

**Keywords:** integrin, CD40L, fibronectin, fibrinogen, high IgM syndrome type 1 (HIGM1), docking simulation, APC, antigen presenting cell, DMEM, Dulbecco's modified Eagle's medium, HIGM1, high IgM syndrome type 1, SLE, systemic lupus erythematosus, TNF, tumor necrosis factor

## Abstract

CD40L is a member of the TNF superfamily that participates in immune cell activation. It binds to and signals through several integrins, including αvβ3 and α5β1, which bind to the trimeric interface of CD40L. We previously showed that several integrin ligands can bind to the allosteric site (site 2), which is distinct from the classical ligand-binding site (site 1), raising the question of if CD40L activates integrins. In our explorations of this question, we determined that integrin α4β1, which is prevalently expressed on the same CD4+ T cells as CD40L, is another receptor for CD40L. Soluble (s)CD40L activated soluble integrins αvβ3, α5β1, and α4β1 in cell-free conditions, indicating that this activation does not require inside-out signaling. Moreover, sCD40L activated cell-surface integrins in CHO cells that do not express CD40. To learn more about the mechanism of binding, we determined that sCD40L bound to a cyclic peptide from site 2. Docking simulations predicted that the residues of CD40L that bind to site 2 are located outside of the CD40L trimer interface, at a site where four HIGM1 (hyper-IgM syndrome type 1) mutations are clustered. We tested the effect of these mutations, finding that the K143T and G144E mutants were the most defective in integrin activation, providing support that this region interacts with site 2. We propose that allosteric integrin activation by CD40L also plays a role in CD40L signaling, and defective site 2 binding may be related to the impaired CD40L signaling functions of these HIGM1 mutants.

CD40L is a type II protein ligand member of the tumor necrosis factor (TNF) superfamily expressed by activated T cells. CD40L is a costimulatory molecule critical in a variety of T cell–antigen presenting cell (APC) interactions, including activating APCs to provide help to cytotoxic T cells (1). It also provides help to B cell to promote class switching (2, 3). In addition to its transmembrane form, CD40L is also released as a soluble ligand (sCD40L) by proteolytic cleavage, allowing it to interact at more distant sites. CD40L appears to modulate other cell types as well (4). CD40L can initiate inflammatory and procoagulatory responses in vascular endothelial cells (5, 6, 7). Findings such as these have led to the belief that CD40–CD40L interactions play a more general role in immune regulation. Given its diverse functions, it is not surprising that CD40L is critical in a variety of chronic autoimmune and inflammatory diseases, including systemic lupus erythematosus (SLE), diabetes, chronic kidney disease (8, 9), among others.

CD40L functions through its interactions with cell surface proteins CD40, and α5β1 and αIIbβ3 integrins. CD40 belongs to the tumor necrosis factor receptor (TNF-R) family and was first identified and functionally characterized on B lymphocytes (10). CD40–CD40L interactions play a more general role in immune regulation and stabilize arterial thrombi through binding to integrin αIIbβ3 (11). αIIbβ3 recognizes the KGD motif at the N terminus of CD40L (residues 115–117 of CD40L). It has also been reported that CD40L binds to integrin α5β1 and transduces signals through this integrin in a CD40 and αIIbβ3-independent manner. Finally, data suggests that CD40 and integrin α5β1 can bind to CD40L simultaneously (12).

We recently identified vascular integrin αvβ3 as a new receptor for CD40L (13). We localized the α5β1 and αvβ3 binding to the trimeric interface of CD40L. CD40L mutants defective in integrin binding in the predicted integrin-binding site were defective in CD40L/CD40 signaling and acted as antagonists of CD40L/CD40 signaling (13). Furthermore, we demonstrated that CD40L binding to αvβ3 activates αvβ3 in an allosteric manner. Of relevance to our discovery is the finding that eight X-Linked Hyper IgM Syndrome (HIGM1)-causative variants have alterations in the CD40L integrin binding, and they are defective in integrin binding and signaling, suggesting that the loss of integrin binding is related to the defect in CD40L signaling in HIGM1. Also, our previous studies found that several proinflammatory integrin ligands (*e.g.*, CX3CL1, CXCL12, and secreted phospholipase A2 type IIA (sPLA-IIA)) activated integrins by binding to a second ligand-binding site (site 2) in an allosteric manner in addition to binding to a primary site (site 1) (14, 15, 16). The recent discovery on the binding of 25-hydroxycholesterol to integrin site 2 upregulates inflammatory cytokines, TNF, and IL-6, production (17) indicates that site-2-mediated integrin activation is involved in proinflammatory signaling.

Herein, we now present data that integrin α4β1 is a new receptor for CD40L. Notably, we showed that CD40L is an allosteric activator of integrins αvβ3, α5β1, and α4β1. Specifically, we demonstrate that CD40L can bind to an allosteric ligand-binding site (Site 2) (14, 15, 16), which is distinct from αvβ3’s classical ligand-binding site (site 1). Also, four HIGM1 mutants are clustered in the site-2-binding site of CD40L, indicating that site-2-binding is potentially involved in CD40L/CD40 signaling. We propose that CD40L acts as a ligand and an allosteric activator of several integrins and mediates proinflammatory signaling independent of CD40. We further propose that this may be a common mechanism for a group of proinflammatory proteins and that CD40L-induced integrin activation is required for CD40L signaling.

## Results

### sCD40L activates soluble integrin αvβ3 in cell-free conditions

**Figure 1:**
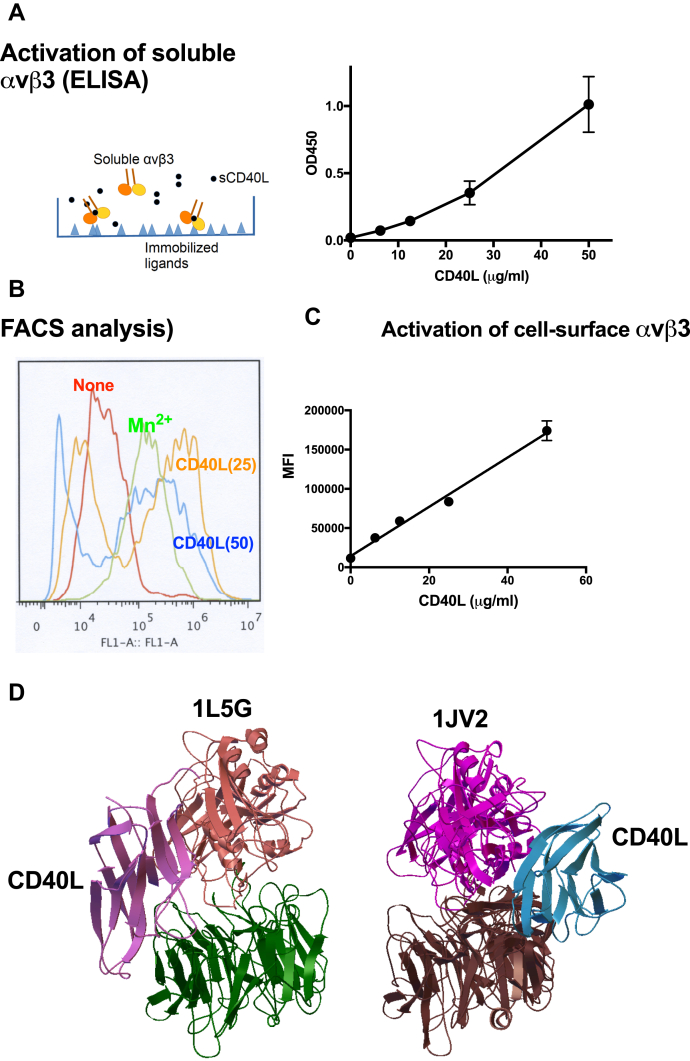
**CD40L activates soluble and cell-surface integrin αvβ3**. *A*, WT soluble CD40L activates soluble integrin αvβ3. Wells of 96-well microtiter plate were coated with γC399tr (50 μg/ml in PBS) for 2 h at room temperature and incubated with soluble αvβ3 (5 μg/ml) and sCD40L for 1 h in HEPES-Tyrodes buffer with 1 mM Ca^2+^ (which keeps αvβ3 in an inactive form). Bound αvβ3 was quantified using anti-β3 antibody (AV10). *B*, WT soluble CD40L activates cell-surface αvβ3. β3-CHO cells were incubated with sCD40L (25 μg/ml) for 30 min on ice in HEPES-Tyrodes buffer with 0.02% BSA and 1 mM Ca^2+^ and then with FITC-labeled γC399tr for 1 h at room temperature. The binding of γC399tr was measured in flow cytometry. A typical flow cytometric profile is shown. *C*, dose dependency of activation of cell surface αvβ3 (MFI, mean fluorescent intensity). *D*, Docking models. We performed docking simulation between CD40L (1AYP.pdb) and inactive αvβ3 using open (1L5G.pdb) or closed headpiece (1JV2.pdb) as a target. Docking model of interaction between αvβ3 (open headpiece, 1L5G.pdb)-CD40L (1AYP.pdb) was taken from (13).

Previous studies showed that several proinflammatory integrin ligands activated integrins in an allosteric manner (see Introductions). We hypothesized that another proinflammatory cytokine CD40L activates integrins. Previous studies showed that soluble integrin αvβ3 bound to many ligands including the fibrinogen γ-chain C-terminal domain with truncation at the C terminus (γC399tr) (See Introduction). γC399tr specifically binds to site 1 of αvβ3, but not to site 2 (14). We studied if sCD40L enhances the binding of soluble αvβ3 to γC399tr. γC399tr was immobilized and incubated with soluble αvβ3 in the presence of 1 mM Ca^2+^ to keep αvβ3 inactive (Fig. 1*A*). sCD40L enhanced binding of soluble αvβ3 to immobilized γC399tr in a dose-dependent manner. Activation is defined by the increase in binding of soluble integrin αvβ3 to immobilized ligand (γC399tr) by soluble activators (sCD40L). These findings suggest that CD40L activates integrin αvβ3 in an allosteric manner.

### sCD40L activates cell-surface integrin αvβ3

We next studied if sCD40L activates integrin αvβ3 on the cell surface using CHO cells (CD40-negative) that express recombinant αvβ3 (β3-CHO cells). β3-CHO cells were incubated with sCD40L and FITC-labeled γC399tr in the assays medium with 1 mM Ca^2+^ to keep αvβ3 inactive and bound FITC-labeled γC399tr was measured in flow cytometry. sCD40L markedly enhanced the binding of γC399tr, indicating that sCD40L activated αvβ3 on the cell surface (Fig. 1*B*). The activation of αvβ3 by sCD40L was dose-dependent (up to 50 μg/ml) and can be detected at 6 μg/ml. The binding of FITC-labeled γC399tr shown by mean fluorescent intensity (MFI) correlated well with the sCD40L concentrations (Fig. 1*D*). Although high concentrations of sCD40L were required to activate soluble integrins, it is likely that integrin activation by sCD40L is biologically relevant, since CD40L is a membrane-bound protein and is highly concentrated on the cell surface.

### CD40L binds to another ligand-binding site (site 2) in integrin αvβ3

We previously showed that several integrin ligands (*e.g.*, CX3CL1, CXCL12, and sPLA2-IIA) bound to an allosteric site (site 2) and activated several integrins (14, 15, 16). We performed docking simulation of interaction between αvβ3 (1JV2.pdb, with closed headpiece) and CD40L monomer (1ALY.pdb) using Autodock3. The simulation predicts that monomeric CD40L binds to site 2 well (docking energy –20.5 kcal/mol) (Fig. 1*D*).

### The site-2-binding interface of sCD40L predicted to be located in the outside of trimer

**Figure 2:**
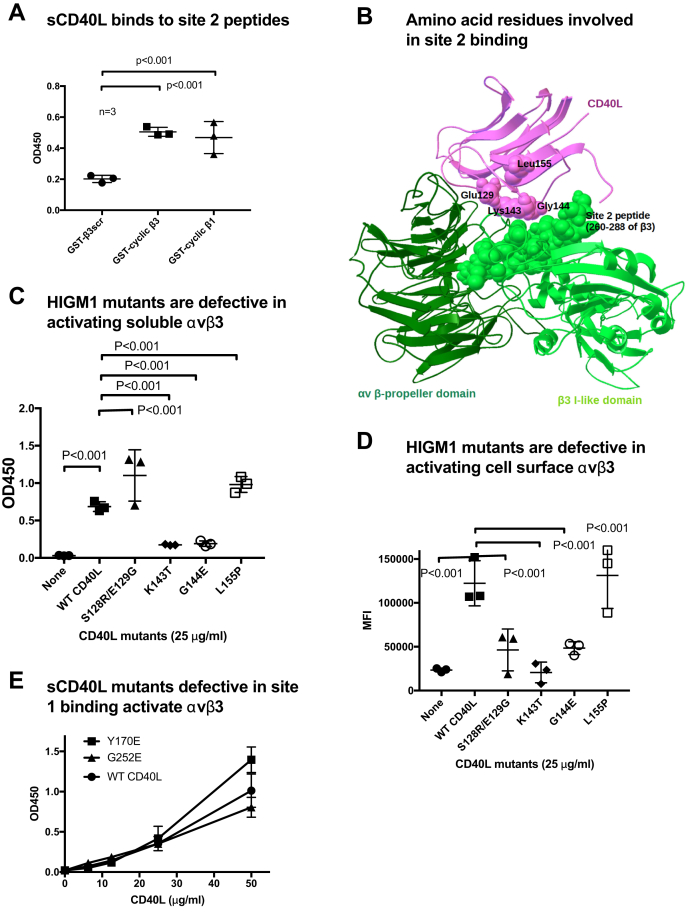
**Localization of CD40L-binding site in integrin αvβ3 and the integrin-binding site in CD40L. *A*, CD40L binds to cyclic site 2 peptide.** CD40L was immobilized and incubated with cyclic site 2 peptides of integrin β1 or β3 subunit fused to GST. Bound GST was measured using HRP-conjugated anti-GST antibodies. Scrambled linear β3 site 2 peptide fused to GST was used as a control. *B*, positions of four HIGM1 mutants in CD40L in the predicted site 2 binding site in CD40L. Docking simulation of CD40L binding to site 2 between CD40L (1ALY.pdb) and the αvβ3 headpiece (1JV2.pdb, closed-headpiece) was performed. Position of site-2-derived peptide in integrin β3 was shown. *C*, activation of soluble integrin αvβ3 by sCD40L HIGM1 mutants in ELISA-type assays. These assays were performed as described in Figure 1 legend. Data are shown as means ± SD (n = 3). *D*, activation of cell-surface integrin αvβ3 by sCD40L HIGM1 mutants by cell binding assays and flow cytometry. These assays were performed as described in Figure 1 legend. Data are shown as means ± SD (n = 3). *E*, CD40L mutants defective in binding to site 1 allosterically activate integrin. The CD40L Y170E and G252E mutants (13), which do not bind to the classical ligand-binding site (site 1), activate soluble integrin αvβ3.

We previously reported that the peptide from site 2 of β3 (QPNDGQSHVGSDNHYSASTTM, residues 267–287 of β3, Cys-273 is changed to S, fused to GST) directly bound to CX3CL1, sPLA2-IIA, and CXCL12 and the peptide suppressed integrin activation by these activators, suggesting that they directly bind to site 2 and mediate integrin activation (14, 15, 16). To prove if CD40L binds to site 2, we tested if CD40L binds to peptides derived from site 2. Linear site 2 peptides did not show good binding to CD40L (not shown). We thus designed disulfide-linked cyclic 28-mer peptides by introducing two Cys residues at both ends to enhance affinity and stability of the peptides. To predict the positions of the two Cys residues that do not affect the peptide conformation of the peptides we used the Disulfide by Design-2 (DbD2) software (18). We found that cyclic site 2 peptide from β3 (C260-RLAGIVQPNDGQSHVGSDNHYSASTTMC288) and the corresponding β1 peptide bound to sCD40L, suggesting that sCD40L binds to site 2 (Fig. 2*A*). The position of cyclic site 2 peptide is shown in Figure 2*B*. These findings suggest that sCD40L activates integrins by binding to site 2.

### Localization of amino acid residues of CD40L involved integrin activation

The docking simulation predicts that four HIGM1 mutations (S128R/E129G, K143T, G144E, and L155P) are clustered in the predicted site-2-binding interface of CD40L (Fig. 2*B*). Amino acid residues involved in the predicted CD40L-αvβ3 (closed form) interaction are shown in Table 1. We studied if these HIGM1 mutations affect CD40L-induced αvβ3 activation. Notably, the K143T and G144E mutants were defective in activating soluble αvβ3 (Fig. 2*C*) and were defective in activating cell surface αvβ3 (Fig. 2*D*). These finding are consistent with the docking model that these mutations (K143T and G144E) are located in the predicted site-2-binding site of CD40L (Fig. 2*B*).

**Table 1:** Amino acid residues involved in the predicted CD40L-αvβ3 (closed form) interaction

CD40L	αV	β3
**Glu129**, Ala130, Ser131, Lys133, Thr134, Thr135, Glu142, **Lys143, Gly144**, Tyr145, Tyr146, **Leu155**, Lys159, Phe177, Cys178, Ser179, Asn180, Arg181, Ala183, Ser184, Pro217, Cys218, Gln220, Pro244, Ser245, Gln246, Val247, Ser248, His249, Gly250, Thr251,	Pro14, Glu15, Asn44, Thr45, Thr46, Gln47, Pro48, Gly49, Ile50, Val51, Glu52, Gln68, Gly76, Asn77, Arg78, Asp79, Ala81, Lys82, Asp83, Asp84, Pro85, Glu87, Phe88, Lys89, Ser90, His91, Arg122,	Met165, Ser168, Glu171, Glu174, Asn175, Pro186, Asp278, His280, Tyr281, Ser282, Ala283, Ser284, Thr285, Thr286

The amino acid residues in CD40L that are mutated in HIGM1 are in bold.

The amino acid residues in β3 that are involved in site 2 peptide are underlined.

Previous studies identified amino acid residues in the trimeric interface that are involved in binding to the classical ligand-binding site (site 1) of activated integrins (13). Consistently, the two CD40L mutations Y170E and G252E in the trimeric interface are defective in binding to site 1 (13), but activated soluble αvβ3 (Fig. 2*E*). This finding is consistent with the model that site-1-binding and site-2-binding sites are distinct.

### sCD40L activates soluble and cell-surface integrin α5β1

Previous studies showed that integrin α5β1 binds to CD40L and induces signals independent of CD40 (12). We previously identified the binding site for α5β1 in the trimeric interface of CD40L, indicating that α5β1 and αvβ3-binding sites overlap in the trimeric interface of CD40L (13). We hypothesized that CD40L activates α5β1 as well in an allosteric manner.

**Figure 3:**
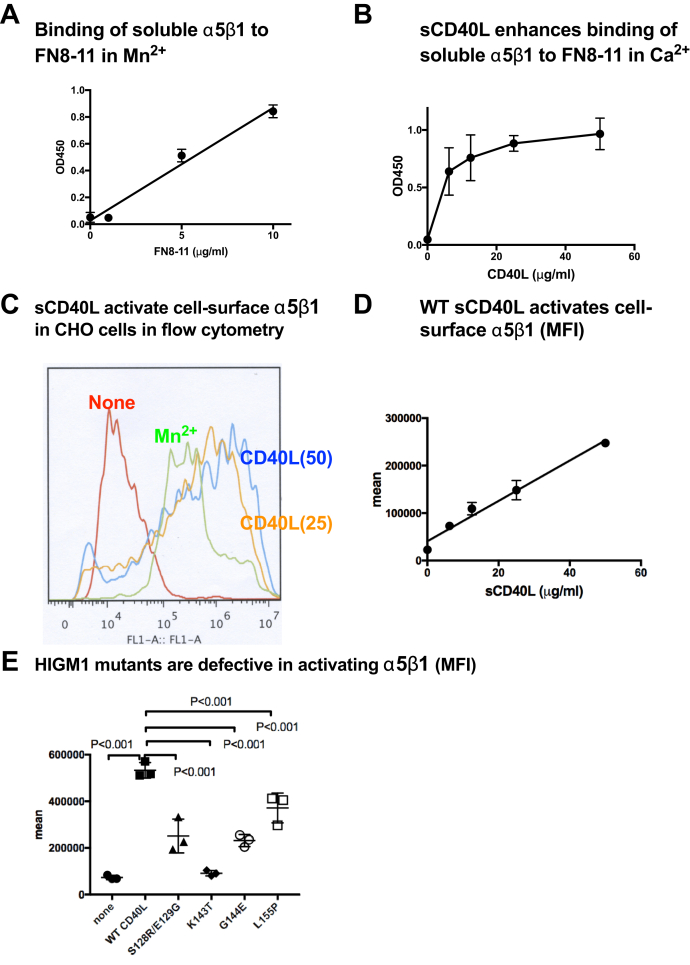
**sCD40L activates soluble and cell-surface α5β1**. *A*, biotinylated soluble α5β1 binds to the fibronectin cell-binding fragment (FN8–11). The fibronectin domains 8–11 conjugated to GST was coated to wells of 96-well microtiter plate. Remaining protein-binding sites were blocked with BSA. Soluble biotinylated soluble α5β1 (1 μg/ml) in 50 μl Tyrode-HEPES buffer with 1 mM Mn^2+^ was added and incubated for 1 h at 37 °C. Bound α5β1 was quantified using HRP-conjugated streptavidin. Data are shown as means ± SD (n = 3). *B*, WT sCD40L activates soluble integrin α5β1. Wells of 96-well microtiter plate were coated with FN8-11 (50 μg/ml in PBS) for 2 h at room temperature and incubated with biotinylated soluble α5β1 (1 μg/ml) and sCD40L for 1 h in HEPES-Tyrodes buffer with 1 mM Ca^2+^ (which keeps α5β1 in an inactive form). Bound α5β1 was quantified using HRP-conjugated streptavidin. *C*, activation of cell-surface α5β1 in CHO cells. This assay was performed as described in Figure 1 legend, except that CHO cells and FITC-FN8-11 were used. The data were analyzed using FlowJo. A typical flow cytometric profile is shown. *D*, dose dependency of activation of cell surface α5β1 (mean fluorescent intensity). This assay was performed as described in Figure 1 legend. Data are shown as means ± SD (n = 3). *E*, activation of cell-surface integrin α5β1 by sCD40L HIGM1 mutants by cell binding assays and flow cytometry. These assays were performed as described in Figure 1 legend. Data are shown as means ± SD (n = 3).

We confirmed that biotinylated soluble α5β1 binds to the fibronectin fragment (FN8-11), a specific ligand to α5β1, in ELISA-type activation assays in the presence of 1 mM Mn^+2^ (Fig. 3*A*). FN8-11 binds to site 1, but does not bind to site 2 (14). We studied if sCD40L activates soluble α5β1. Activation is defined by the increase in integrin binding to immobilized FN8-11 by soluble sCD40L. FN8-11 was immobilized and incubated with biotinylated soluble α5β1 in the presence of 1 mM Ca^2+^. sCD40L enhanced the binding of soluble α5β1 to immobilized FN8-11 in a dose-dependent manner (Fig. 3*B*). These findings indicate that sCD40L activates soluble integrin α5β1 in cell-free conditions.

We showed that sCD40L activated cell-surface α5β1 in CHO cells (α5β1+, CD40-) using FITC-labeled FN8-11 in a dose-dependent manner (Fig. 3*B*). The levels of α5β1 activation by sCD40L in MFI and sCD40L concentrations correlated well (Fig. 3, *C* and *D*). We studied if four HIGM1 mutants can activate cell-surface α5β1 in CHO cells. They were defective in activating α5β1 and the K143T was the most defective (Fig. 3*E*). These findings suggest that sCD40L binds to site 2 of α5β1 in a manner similar to that of αvβ3 and activates α5β1.

### sCD40L activates soluble and cell-surface integrin α4β1

**Figure 4:**
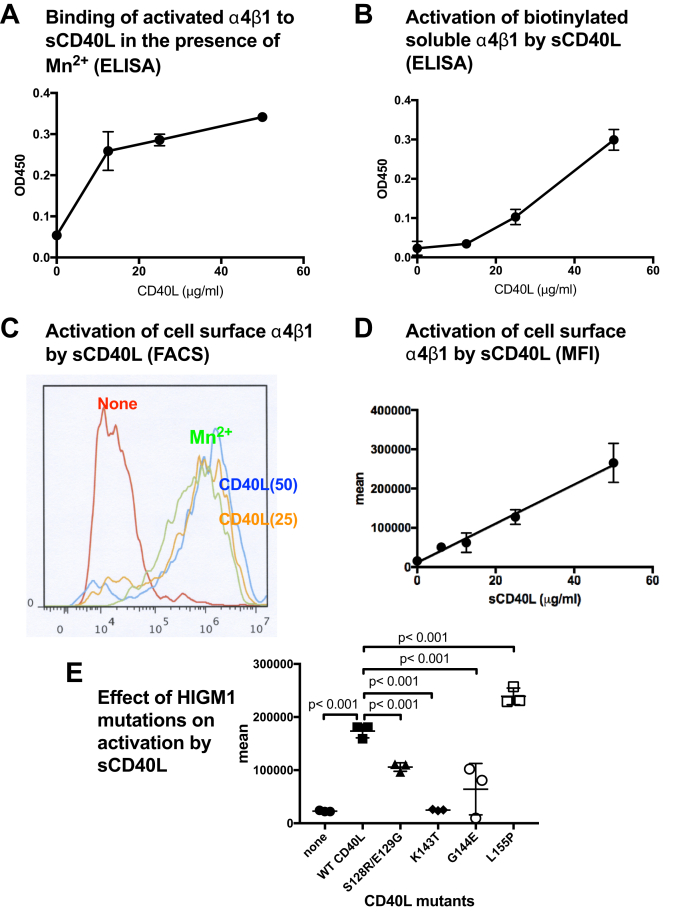
**sCD40L activates soluble and cell-surface α4β1**. *A*, WT sCD40L activates soluble integrin α4β1**.** Wells of 96-well microtiter plate were coated with H120 (50 μg/ml in PBS) for 2 h at room temperature and incubated with biotinylated soluble α4β1 (1 μg/ml) and sCD40L for 1 h in HEPES-Tyrodes buffer with 1 mM Ca^2+^ (which keeps α4β1 in an inactive form). Bound α4β1 was quantified using HRP-conjugated streptavidin. Data are shown as means ± SD (n = 3). *B*, WT sCD40L activates soluble integrin α4β1**.** Wells of 96-well microtiter plate were coated with FN8-11 (50 μg/ml in PBS) for 2 h at room temperature and incubated with biotinylated soluble α5β1 (1 μg/ml) and sCD40L for 1 h in HEPES-Tyrodes buffer with 1 mM Ca^2+^ (which keeps α5β1 in an inactive form). Bound α5β1 was quantified using HRP-conjugated streptavidin. *B*, WT sCD40L activates soluble integrin α4β1**.** Wells of 96-well microtiter plate were coated with H120 (50 μg/ml in PBS) for 2 h at room temperature and incubated with biotinylated soluble α4β1 (1 μg/ml) and sCD40L for 1 h in HEPES-Tyrodes buffer with 1 mM Ca^2+^ (which keeps α4β1 in an inactive form). Bound α4β1 was quantified using HRP-conjugated streptavidin. *C*, WT sCD40L activates cell-surface α4β1. α4-CHO cells were incubated with sCD40L (25 μg/ml) for 30 min on ice in HEPES-Tyrodes buffer with 0.02% BSA and 1 mM Ca^2+^ and then with FITC-labeled H120 for 1 h at room temperature. The binding of H120 was measured in flow cytometry. A typical flow cytometric profile is shown. *D*, dose dependency of activation of cell surface α4β1 (mean fluorescent intensity). Data are shown as means ± SD (n = 3). *E*, activation of cell-surface integrin α4β1 by sCD40L HIGM1 mutants by cell binding assays and flow cytometry. These assays were performed as described in Figure 1 legend. Data are shown as means ± SD (n = 3).

CD40L is primarily expressed in activated CD4+ T cells, but αvβ3 and α5β1 are not major integrins in T cells. sCD40L binds to site 1 of activated αvβ3 and α5β1 (13). It is unclear if integrin α4β1, which is expressed primarily in immune-competent cells, interacts with CD40L. We used biotinylated soluble α4β1 to study if α4β1 binds to CD40L. We found that soluble α4β1 activated by 1 mM Mn^2+^ bound to immobilized WT sCD40L in a dose-dependent manner (Fig. 4*A*), indicating that CD40L is a ligand for α4β1 that binds to site 1.

We then studied if sCD40L activates α4β1 by binding to site 2. A fibronectin fragment H120, a ligand specific to α4β1, binds to site 1, but does not bind to site 2 (14). Activation is defined by the increase in the binding of soluble integrin α4β1 to immobilized H120 by sCD40L. We found that sCD40L enhanced the binding of soluble α4β1 to H120 in a dose-dependent manner in the presence of 1 mM Ca^2+^ (Fig. 4*B*).

Also, sCD40L enhanced the binding of FITC-labeled H120 to CHO cells that express recombinant α4β1 (α4-CHO cells) in the presence of 1 mM Ca^2+^ (Fig. 4*C*). The α4β1 activation (MFI) correlated well with the sCD40L concentrations, indicating that sCD40L activated cell-surface α4β1 (Fig. 4*D*). We studied if the four HIGM1 mutants that are clustered in the predicted site-2-binding site of CD40L can activate α4β1 on α4-CHO cells. The HIGM1 mutants were defective in activating cell surface α4β1. The K143T and G144E mutants were the most defective in activating α4β1 among four HIGM1 mutants (Fig. 4*E*), indicating that site-2-binding interface in α4β1 is similar to those of αvβ3 and α5β1. These findings suggest that α4β1 is a new receptor for CD40L. CD40L binds to site 2 and activates α4β1 in a manner similar to those of αvβ3 and α5β1.

### Characteristics of four HIGM1 mutants

**Figure 5:**
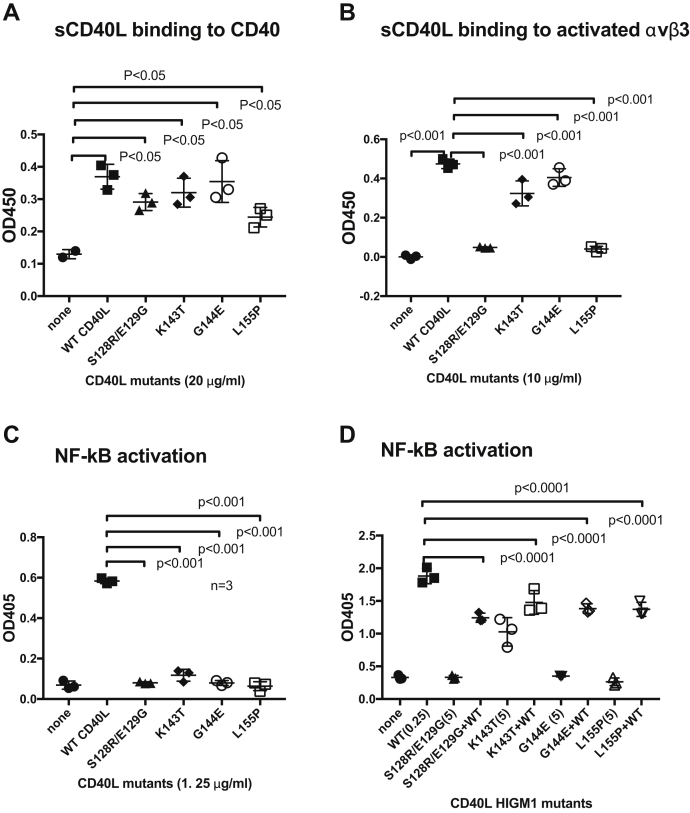
**Four HIGM1 mutants clustered in the predicted site-2-binding region of CD40L are defective insignaling and act as antagonists**. *A*, binding of the CD40 fragment fused to GST to CD40L (WT and HIGM1 mutants). The CD40 fragment (residues 21–144) fused to GST (100 μg/ml in PBS) was immobilized to wells of a 96-well microtiter plate and incubated with sCD40L mutants. Bound sCD40L mutants were measured using anti-histidine Abs. Data are shown as means ± SD (n = 3). *B*, binding of sCD40L to site 1 of soluble integrin αvβ3 in the presence of 1 mM Mn^2+^. sCD40L (residues 118–261) was immobilized to wells of a 96-well microtiter plate and incubated with soluble αvβ3 (5 μg/ml) in HEPES-Tyrodes buffer (1 mM Mn^2+^). Bound αvβ3 was measured using anti-β3 (AV10). Data are shown as means ± SD (n = 3). *C*, NF-kB activation by sCD40L (WT and HIGM1 mutants). HEK293 cells that express CD40 and NF-kB reporter gene (SEAP) were incubated with sCD40L in serum-free Dulbecco's modified Eagle's medium for 4 h, and SEAP in the medium was determined. Data are shown as means ± SD (n = 3). *D*, suppression of NF-kB activation by HIGM1 mutants. NF-KB activation was measured in reporter cells as described in (*C*). WT CD40L (0.25 μg/ml) and mutants (5 μg/ml) were used. Data are shown as means ± SD (n = 3).

We further characterized the four HIGM1 mutants to determine the role of site 2 in CD40L signaling. They all bound to CD40 in ELISA (Fig. 5*A*). We tested their ability to bind to activated soluble αvβ3 in ELISA in the presence of 1 mM Mn^2+^, which reflects their ability to bind to site 1. S128R/E129G and L155P were defective and K143T and G144E were partially defective in binding to activated integrin αvβ3, suggesting that K143T and G144E are still able to bind to site 1 (Fig. 5*B*). S128R/E129G and L155P did not bind to activated αvβ3 (to site 1) although these mutations are not in the site-1-binding site in the trimeric interface. It is possible that these mutations induced conformational changes in the trimeric interface.

We determined the ability of HIGM1 mutants to induce NF-kB activation in HEK293 reporter cells. The four mutants were all defective in inducing NF-kB activation in reporter cells, except that K143T is slightly active (Fig. 5*C*). K143T at high concentration (5 μg/ml) induced substantial NF-kB activation (Fig. 5*D*). We tested if excess (20-fold) mutants can suppress NF-kB activation by WT sCD40L. We found that all mutants tested suppressed NF-kB activation by WT sCD40L (Fig. 5*D*), indicating that they act as antagonists. We previously showed that eight other HIGM1 mutants, which are clustered in the trimeric interface, were defective in integrin binding (to site 1). Their defect in site 1 binding is likely related to their defect in CD40L signaling (13). The two mutants, K143T and G144E, still bind to site 1 but defective in integrin activation by binding to site 2. We thus propose that the binding of CD40L to site 2 is required for NF-kB activation.

## Discussion

### Potential biological role of allosteric integrin activation by CD40L

**Figure 6:**
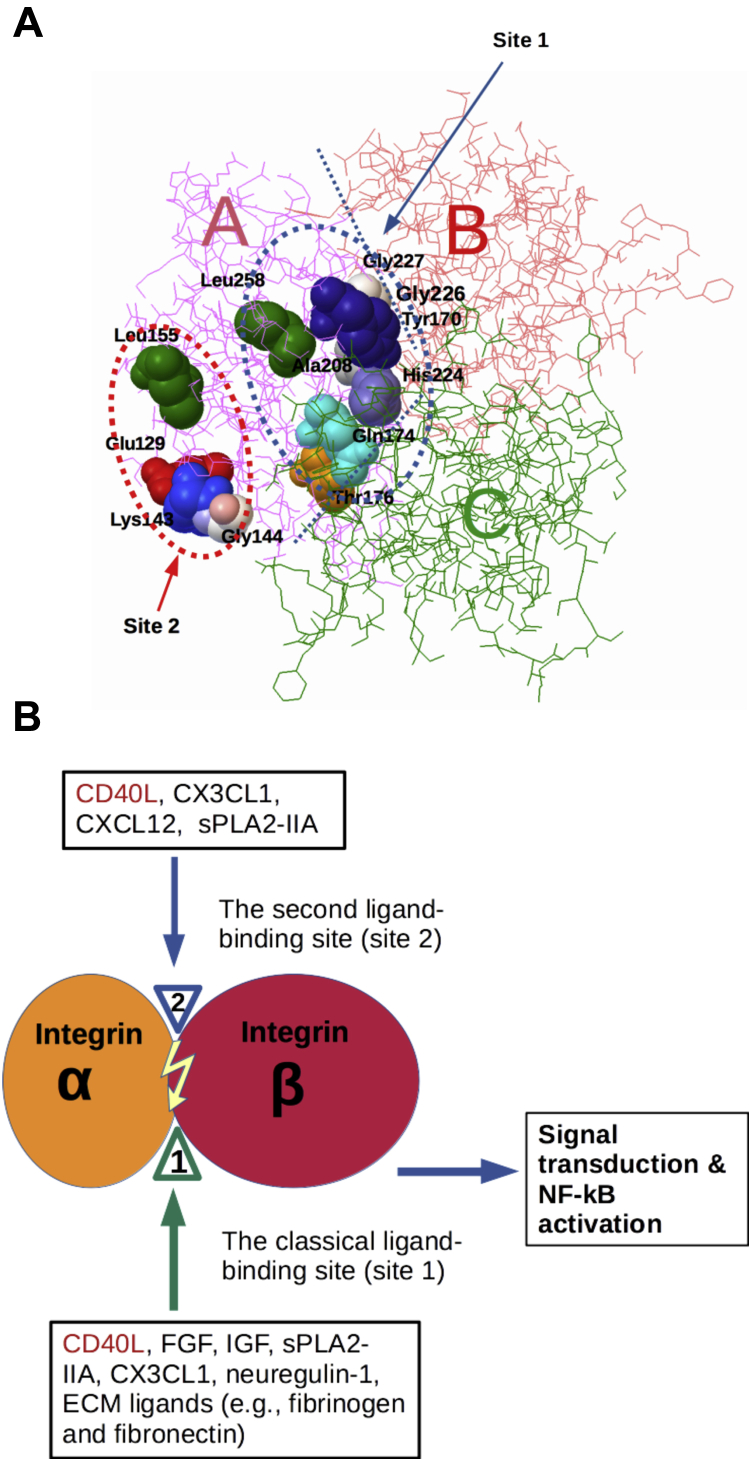
**CD40L integrin interaction and its potential roles**. *A*, clustering of HIGM1 mutants in site 1 and site-2-binding sites in CD40L. Previous study showed that eight HIGM1 mutants are clustered in the trimeric interface of CD40L. The present study shows that four HIGM1 mutants are clustered in the site-2-binding site of CD40L in the outside of the CD40L trimer. *B*, integrin ligands that bind to site 1 and site 2. We identified the second ligand-binding site (site 2) by docking simulation of ligand binding using inactive integrin as a target. CD40L binding to site 2 enhances ligand binding to site 1of integrins. The ligand specificity to site 2 overlaps with that of site 1, but ECM ligands do not appear to bind to site 2.

In the present study, we showed that sCD40L activated soluble and cell-surface integrins αvβ3, α5β1, and α4β1 in a dose-dependent manner. These findings indicate that this activation does not require inside-out signaling. Since cyclic site 2 peptide bound to sCD40L, it is suggested that sCD40L bound to site 2. Docking simulation using inactive/close headpiece αvβ3 as a target predicts that CD40L binds to site 2 and four HIGM1 mutants are clustered in the predicted site-2-binding site in CD40L (Fig. 6*A*). We showed that the HIGM1 mutants (particularly K143T and G144E) are defective in activating integrins, consistent with the prediction. The present study showed that sCD40L is a new allosteric activator of integrins by binding to site 2. The four HIGM1 mutants, including K143T and G144E, were defective in activating integrins αvβ3, α5β1, and α4β1 because they are defective in site 2 binding. This suggests that the defective binding of CD40L to site 2 is related to defective CD40L/CD40 signaling. Since integrins on normal blood cells (*e.g*., B cells) are not activated, it is possible that CD40L-mediated integrin activation by binding to site 2 is required for CD40L/CD40 signaling, in addition to site 1 binding. Previous study showed that eight HIGM1 mutants clustered in the trimeric interface are defective in integrin binding and in signaling because they are defective in site 1 binding (13), indicating that the defect in integrin binding to site 1 is related to defective CD40L/CD40 signaling. We thus propose that CD40L-mediated integrin activation by binding to site 2 is also required for CD40L signaling. Since integrins are not activated in normal leukocytes, integrin activation by this mechanism will facilitate signaling by CD40L.

It is well known that high concentrations of sCD40L (>1000-fold) are required to induce CD40L/CD40 signaling compared with membrane-bound CD40L. In our previous study, sCD40L at >1 μg/ml was needed to achieve maximal NF-kB activation in HEK293 reporter cells (13). We proposed that CD40L/CD40 signaling requires direct integrin binding to CD40L (and subsequent integrin-CD40L-CD40 ternary complex formation). Integrins are not high-affinity receptors compared with CD40. This partly explains that high concentrations of sCD40L are required for CD40L/CD40 signaling. In the present study, very high concentrations of sCD40L were required to detect integrin activation. It is unlikely that integrin activation can be induced by sCD40L under physiological conditions even in autoimmune disease patients (*e.g.*, SLE). We thus propose that integrin activation is primarily induced by membrane-bound CD40L in activated T cells or platelets, in which CD40L is brought to the cell surface upon activation of T cells or platelets, and CD40L is highly concentrated on the cell surface. It is, however, possible that sCD40L can be concentrated by binding to cell surface CD40. If so, sCD40L at levels in autoimmune disease patients may sufficiently trigger integrin activation and subsequent CD40/CD40L signaling for inflammatory responses. The present results provide the first evidence that CD40L can induce integrin activation, regardless of whether it is soluble or membrane-bound form.

The binding of CD40L to site 2 of integrins may lead to proinflammatory outside-in signaling, as shown by the binding of 25-hydroxycholesterol to site 2 (17). It is possible that integrin activation by binding of CD40L to site 2 will facilitate other growth factor/cytokine signaling, since direct integrin binding of growth factors/cytokines/proinflammatory proteins to the classical ligand-binding site (site 1) (Fig. 6*B*) is required for their signaling functions. This includes FGF-1 (19), IGF-1 (20), and neuregulin-1 (21), CX3CL1(22), IL-1β (23), CD40L (13), and sPLA2-IIA (24). It is imperative to study the role of allosteric integrin activation in growth factor/cytokine signaling in future studies.

### Integrin activation by inside-out signaling versus integrin activation by site 2 binding

It has been generally accepted that integrins are activated by inside-out signaling. Integrin activation is accompanied by global conformational changes (25, 26). It has been, however, reported that inside-out signaling enhanced clustering of integrin αIIbβ3 and global conformational changes in thrombin-activated platelets but did not enhance ligand-binding affinity to monovalent ligand (27). Also, inside-out signaling induced by cross-linking of T cell receptor did not enhance ligand-binding affinity of leukocyte integrins to monovalent ligand (28). It is possible that inside-out signaling does not enhance ligand affinity (the integrin headpiece is still in close conformation). The present study showed that sCD40L enhanced ligand-binding affinity of integrins to monomeric ligands by binding of CD40L to site 2 in the extracellular milieu. Previous studies showed that CX3CL1 (14), CXCL-12 (16), and sPLA2-IIA (15) activated integrins by binding to site 2 in the absence of inside-out signaling. We propose that site-2-mediated integrin activation and inside-out signaling occur independently and complement to each other.

## Experimental procedure

### Materials

Recombinant soluble αvβ3 was synthesized in Chinese hamster ovary (CHO) K1 cells using the soluble αv and β3 expression constructs and purified by Ni-NTA affinity chromatography, as described (29). Biotinylated soluble α5β1 and α4β1 were obtained from Acro Biosystems. β3-CHO and α4-CHO cells have been described (24). Fibrinogen γ-chain C-terminal domain that lacks residues 400–411 (γC399tr) was synthesized as described (30). GST-fusion proteins of fibronectin type III domains 8–11 (FN8-11) and fibronectin H120 fragment (FN-H120) were described (22). Anti-human β3 mAb AV10 was provided by B. Felding (The Scripps Research Institute, La Jolla, CA). HRP-conjugated anti-His tag antibody was purchased from Qiagen (Valencia, CA).

### Synthesis of recombinant sCD40L with no KGD motif

We synthesized recombinant sCD40L (residues 118–261, QNPQIAAHVISEASSKTTSVLQWAEKGYYTMSNNLVTLENGKQLTVKRQGLYYIYAQVTFCSNREASSQAPFIASLCLKSPGRFERILLRAANTHSSAKPCGQQSIHLGGVFELQPGASVFVNVTDPSQVSHGTGFTSFGLLKL) with no N-terminal KGD motif as described (13). sCD40L mutants were generated by site-directed mutagenesis of wild-type (WT) sCD40L (residues 118–261) and synthesized as described for WT sCD49L.

### Synthesis of CD40 fused to GST

The cDNA fragment encoding the CD40 fragment (residues 21–144) was amplified by PCR and subcloned into the BamHI/EcoRI site of PGEX2T. We synthesized the proteins in BL21 cells and purified using glutathione-Sepharose affinity chromatography.

### Binding of the CD40 fragment fused to GST to CD40L

The CD40 fragment fused to GST was coated to wells of a 96-well microtiter plate (100 μg/ml in PBS) for 1 h, and the remaining protein-binding sites were blocked by BSA (0.1%).We then incubated the wells with sCD40L and incubated for 1 h, and bound sCD40L was measured using HRP-conjugated anti-His.

### Synthesis of cyclic site 2 peptides

We introduced 6His tag to the BamHI site of pGEX-2T (resulting vector is designated pGEX-2T6His). We synthesized GST fusion protein of site 2 peptide (QPNDGQSHVGSDNHYSASTTM, residues 267–287 of β3, C273 is changed to S) and a scrambled site 2 peptide (VHDSHYSGQGAMSDNTNSPQT) by subcloning oligonucleotides that encodes these sequences into the BamHI/EcoRI site of pGEX-2T6His as described (14, 15). We introduced a disulfide linkage that connects both ends of the site 2 peptide without affecting its conformation using Disulfide by Design-2 (DbD2) software (http://cptweb.cpt.wayne.edu/DbD2/) (18). It predicted that mutating Gly260 and Asp288 to Cys disulfide-linked cyclic site 2 peptide of β3 does not affect the conformation of the peptide. We generated C260-RLAGIVQPNDGQSHVGSDNHYSASTTMC288, 29-mer cyclic β3 peptide. We designed the corresponding cyclic β1 peptide (C268-KLGGIVLPNDGQSHLENNMYTMSHYYC295, 28-mer cyclic β1 peptide) in which C281 is converted to S. We synthesized the proteins in BL21 cells and purified using glutathione-Sepharose affinity chromatography.

### Binding of soluble integrins to ligands

ELISA-type binding assays were performed as described previously (22). Briefly, wells of 96-well Immulon 2 microtiter plates (Dynatech Laboratories, Chantilly, VA) were coated with 100 μl PBS containing γC399tr for 2 h at 37 °C. Remaining protein-binding sites were blocked by incubating with PBS/0.1% BSA for 30 min at room temperature. After washing with PBS, soluble recombinant αvβ3 (5 μg/ml) in the presence or absence of CD40L (WT or mutant) was added to the wells and incubated in HEPES-Tyrodes buffer (10 mM HEPES, 150 mM NaCl, 12 mM NaHCO_3_, 0.4 mM NaH_2_PO_4_, 2.5 mM KCl, 0.1% glucose, 0.1% BSA) with 1 mM CaCl_2_ for 1 h at room temperature. After unbound αvβ3 was removed by rinsing the wells with binding buffer, bound αvβ3 was measured using anti-integrin β3 mAb (AV-10) followed by HRP-conjugated goat anti-mouse IgG and peroxidase substrates. For activation assays with α5β1, biotinylated soluble α5β1 and α4β1(AcroBio) and their specific ligands (FN8-11 and H120, respectively) were used, and bound biotinylated soluble integrins were measured by using streptavidin conjugated to HRP.

### Flow cytometry

CHO cells were cultured in Dulbecco's modified Eagle's medium (DMEM)/10% fetal calf serum. The cells were resuspended with HEPES-Tyrodes buffer/0.02% BSA (heat-treated at 80 C for 20 min to remove contaminating cell adhesion molecules). The β3-CHO or CHO cells were then incubated with WT or mutant CD40L for 30 min on ice and then incubated with FITC-labeled integrin ligands (γC399tr, FN8-11 and H120) for 30 min at room temperature. The cells were washed with PBS/0.02% BSA and analyzed by BD Accuri flow cytometer (Becton Dickinson, Mountain View, CA). The data were analyzed using FlowJo 7.6.5.

### CD40L reporter assays

We used HEK293 cells that express human CD40L-soluble embryonic alkaline phosphatase (SEAP) reporter (Invivogen). Cells were maintained in DMEM/10% fetal calf serum. For reporter assays, cells were plated in wells of a 24-well culture plate (2.5 × 10^5^ cells in 500 μl DMEM without fetal calf serum) and stimulated with CD40L for 4 h. We measured the levels of alkaline phosphatase activity in the medium, as described (31).

### Docking simulation

Docking simulation of interaction between CD40L (Protein Data Bank code 1ALY), which does not contain the N-terminal KGD motif, and integrin αvβ3 was performed using AutoDock3, as described (24). In the current study, we used the headpiece (residues 1–438 of av and residues 55–432 of β3) of αvβ3 (closed-headpiece form, Protein Data Bank code 1JV2). Cations were not present in αvβ3 during docking simulation (15, 16).

### Statistical analysis

Treatment differences were tested using ANOVA using Prism 7 (GraphPad Software).

## Data availability

Coordinate of the docking model presented in this paper is available upon request. All remaining data are contained within the article.

## Conflict of interest

The authors declare that they have no conflicts of interest with the contents of this article.
